# Alterations in looking at face-pareidolia images in autism

**DOI:** 10.1038/s41598-025-98461-7

**Published:** 2025-04-28

**Authors:** Jessica Galli, Marika Vezzoli, Erika Loi, Serena Micheletti, Anna Molinaro, Lucia Tagliavento, Stefano Calza, Alexander N. Sokolov, Marina A. Pavlova, Elisa Fazzi

**Affiliations:** 1 Department of Clinical and Experimental Sciences, University of Brescia, Brescia, Italy; 2 Unit of Child Neurology and Psychiatry, ASST Spedali Civili of Brescia, Brescia, Italy; 3 BDbiomed, BODaI Lab, University of Brescia, Brescia, Italy; 4 Unit of Biostatistics and Bioinformatics, Department of Molecular and Translational Medicine, University of Brescia, Brescia, Italy; 5 Social Neuroscience Unit, Department of Psychiatry and Psychotherapy, Tübingen Center for Mental Health (TüCMH), Medical School and University Hospital, Eberhard Karls University of Tübingen, 72076 Tübingen, Germany

**Keywords:** Autism spectrum disorder, Face pareidolia, Eye tracking, Visual scanning, Social cognition, Neuroscience, Psychology, Neurology

## Abstract

Face tuning is vital for adaptive and effective social cognition and interaction. This capability is impaired in a wide range of mental conditions including autism spectrum disorder (ASD). Yet the origins of this deficit are largely unknown. Here, an eye-tracking methodology had been implemented in adolescents with high-functioning ASD and in typically developing (TD) matched controls while administering a face-pareidolia task. The spatial distributions of eye fixation in five regions of interest [face, eyes, mouth, CFA (complementary face area, a face area beyond eyes and mouth) and non-face area (a screen area outside a face)] were recorded during spontaneous recognition of a set of Arcimboldo-like Face-n-Food images presented in a predetermined order from the least to most resembling a face. Individuals with ASD gave significantly fewer face responses and looked more often at the mouth, CFA, and non-face areas. By contrast, TD controls mostly fixated the face and eyes areas. The atypical visual scanning strategies could, at least partly, account for the lower face tuning in ASD, supporting the eye avoidance hypothesis, according to which ASD individuals concentrate less on the eyes because the eyes represent a source of emotional information that may make them feel uncomfortable.

## Introduction

Alterations in social cognition along with communication deficits, restricted interests as well as repetitive and stereotyped patterns of behavior are considered a core feature of autism spectrum disorder (ASD)^1,2^. Social cognition deficits may range from a complete lack of interest in engaging with others to subtle difficulties in social interaction^3^. The origins of social impairment in ASD have been intensively investigated using depictions of *real* faces^4,5^. Face processing and recognition are considered to be foundational to social cognition^6–9^. Even young typically developing (TD) infants promptly develop face recognition skills and learn to detect gaze direction and expressions of emotions already within the first year of life^10^. Preference for face-like patterns (eliciting face pareidolia – seeing faces in non-face images) and the ability to recognize certain facial properties arises early in healthy development^11–13^ and is mediated by ancient subcortical pathways^14^.

Mounting evidence indicates that individuals with ASD are impaired in processing and recognition of face-pareidolia images^15–18^ that points to a lower sensitivity to the face schema. However, the origin of this deficit is unclear. Several possible accounts for the lower face tuning in face-pareidolia images in ASD may be considered. First, individuals with ASD have been suggested to preferably use the feature-based rather than holistic face-processing strategies. In other words, they are more attracted to isolated facial elements (such as a mouth) rather than to a combination of features as a whole or *Gestalt*^19^. However, some authors report that high-functioning children with ASD develop holistic face processing^20^. The second explanation sets a focus on dysfunctions of the neural networks underpinning face recognition in individuals with ASD^21,22^. Indeed, in ASD, face-pareidolia images lead to atypically increased functional magnetic resonance imaging (fMRI) activation of the amygdala as well as face-specific cortical network^17^. These authors suggest that an early imbalance in the excitatory and inhibitory systems in ASD affecting typical brain maturation may lead to over-responsive reactions to face-like images and eye contact. However, a similar pattern of brain activation for both individuals with ASD and TD controls during face recognition tasks is also reported^23^.

The third assumption supported by the sharp increase of eye-tracking studies^5^ is that ASD individuals exhibit atypical face scanning strategies represented by abnormal distribution of gaze fixations^24,25^. Specifically, low interest toward the eyes in ASD is well-known along with the absence of significant differences between ASD and TD in gaze fixation to the mouth region^22,26^. At the same time, there is also considerable evidence documenting similarities in face scanning between ASD patients and TD individuals^27,28^. These differences may be accounted for by the heterogeneity of visual images used (static vs. dynamic, affective vs. neutral, familiar vs. novel, presented in isolation vs. within a social scene, and more vs. less ecologically pertinent)^25,29^.

In light of these premises, the present study was directed at examining possible alterations in visual scanning strategies accompanying face tuning in adolescents with high-functioning ASD as compared with TD controls person-by-person matched for gender and age. For avoiding habitual facial features (such as a nose or a mouth) that implicate face presence, we used the Face-n-Food face-pareidolia images^30–34^. These non-face images consist of food ingredients (fruits and vegetables) that do not automatically trigger face processing. The hypothesis under consideration is that lower face tuning in face-like images in ASD adolescents^16^ is accompanied by atypical distribution of a gaze. The alterations in eye fixation can potentially lead to difficulties in seeing a face in face-pareidolia images.

## Materials and methods

The study was conducted in accordance with the ethical guidelines set forth by the Declaration of Helsinki and was approved by the Institutional Review Board of the ASST Spedali Civili of Brescia, Italy (NP2282). Written informed consent was obtained from all participants and their parents or care-providers prior to the collection of data. Participation was voluntary, and the data were processed anonymously.

### Participants

Sixteen individuals with ASD (15 males, 1 female; mean age, 14.13 ± 1.86 years, range 11–17 years, median 15, 95% confidence interval, CI, 13.21–15.04) recruited from the outpatient Unit of Child and Adolescent Neurology and Psychiatry, ASST Spedali Civili of Brescia, Italy, and 16 TD controls (15 males, 1 female; mean age, 14.56 ± 1.90 years, range 11–17, median 15, 95% CI 13.62–15.49) person-by-person matched for gender and age, and recruited from the local community, were enrolled in the study between January and May 2016. No difference in age [Mann-Whitney test, *U* = 124, *p* = 0.89, n.s., two-tailed] was found between the groups. The same participants were engaged in earlier behavioral work^16^. Inclusion criteria for patients were: ASD diagnosis, full intelligence quotient (FIQ) > 70 standard scores (s.s.), verbal intelligence quotient (VIQ) > 70 s.s., absence of genetic conditions, history of brain injury or comorbid mental disorders. The diagnosis of autism was made by an independent clinician according to the Diagnostic and Statistical Manual of Mental Disorders, 5th edition (DSM-V)^2^ and supported by the Autism Diagnostic Interview-Revised (ADI-R)^35^ and the Autistic Diagnostic Observation chedule-II edition (ADOS-2)^36^. Twelve adolescents (75%) had severity level 1 (DSM-5), 3 (19%) had level 2, and the only female ASD patient involved in the study had level 3 (6%). The FIQ and VIQ of ASD individuals was evaluated using the Wechsler Scales of Intelligence for Children-IV edition (WISC-IV)^37^: FIQ mean score was 100.06 ± 13.08 (range 78–128) and VIQ mean score was 100.31 ± 12.35 (range 76–128). TD adolescents attended the mainstream school and, according to school and parents’ reports, did not exhibit cognitive or social impairments in everyday life.

### Materials

#### Stimuli and design

**Fig. 1:**
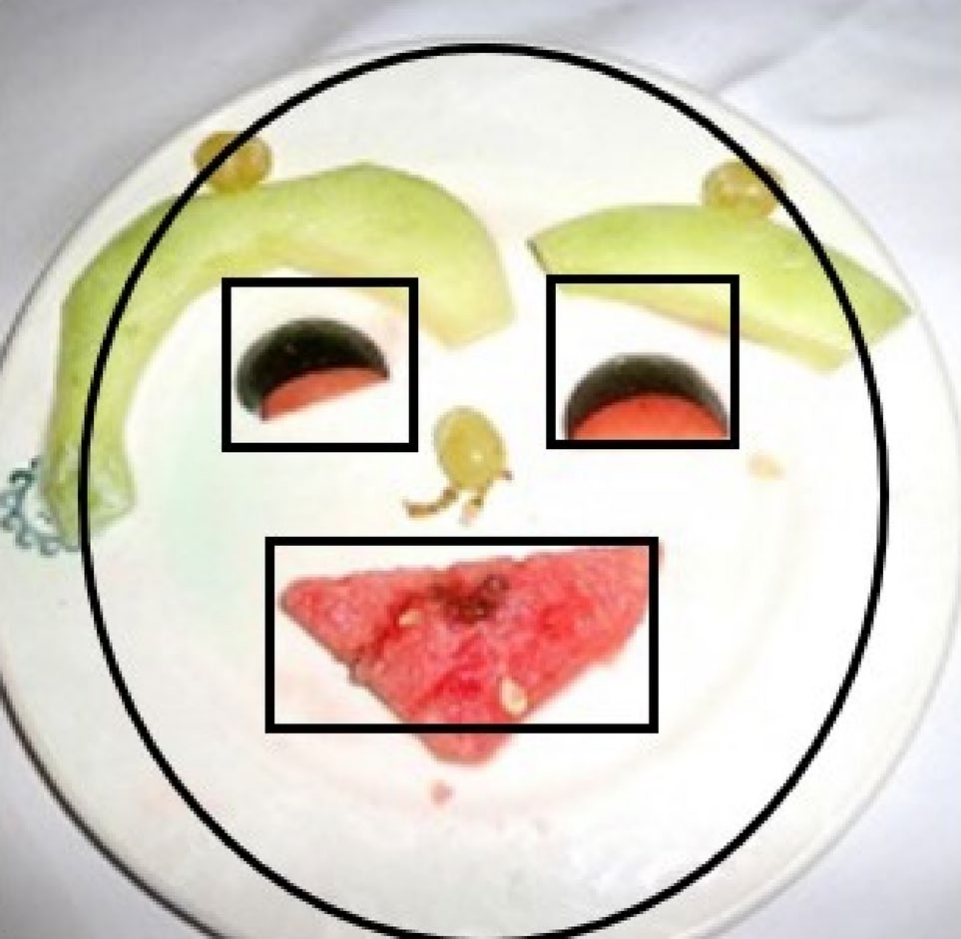
Regions of interest (ROIs) in the image, positioned to resemble the distributions of the features of a human face: a face was represented as an ellipse of 943,942-pixel area, containing a rectangular mouth of 12,180-pixel area, and the right and left eyes, rectangles of respectively 5548 and 5175 pixels.

**Fig. 2:**
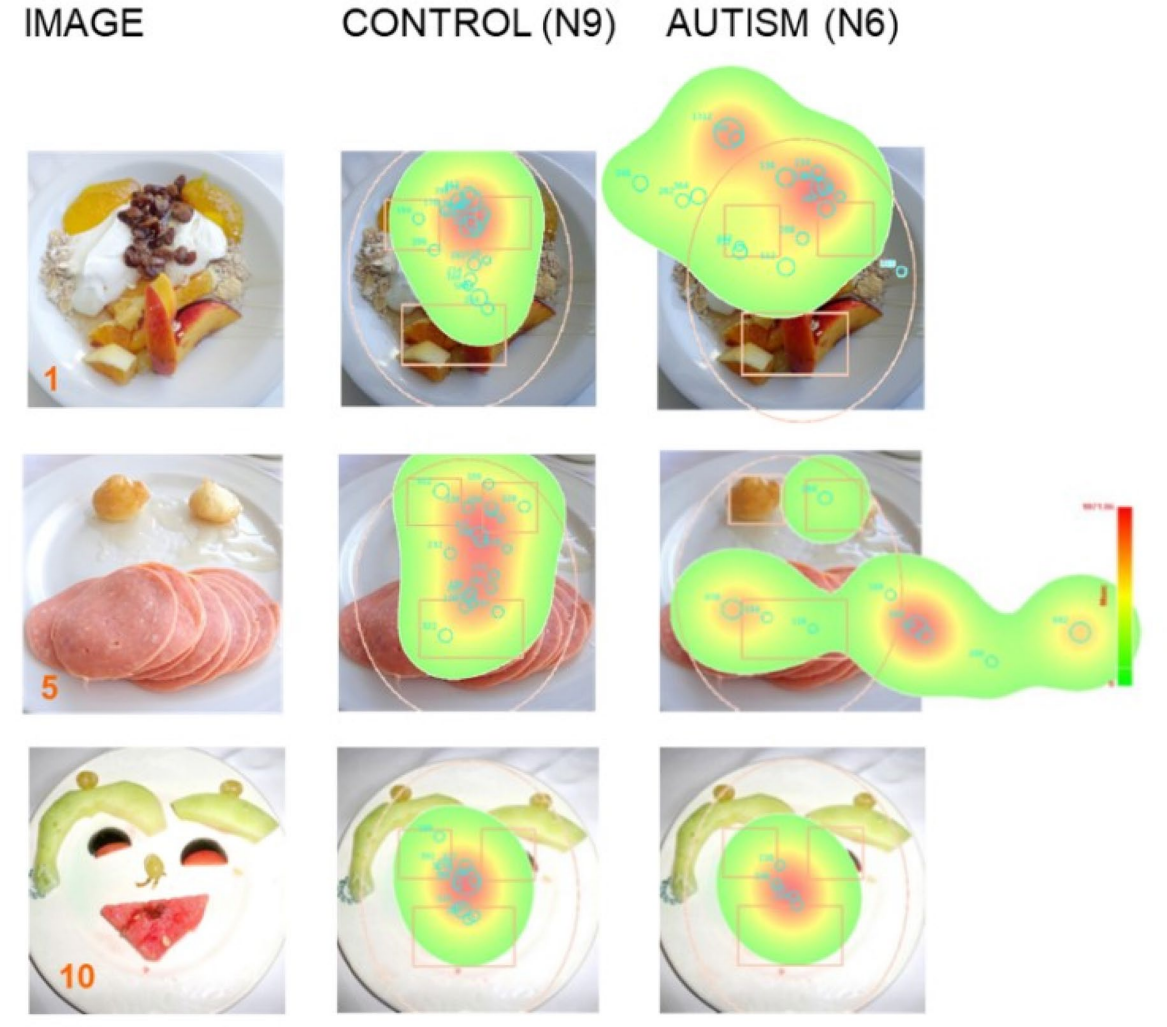
Visual fixation maps in a participant with ASD (N6) and in a TD (N9) control for images 1, 5, and 10. For image 5, the proportion of time spent looking at a face is greater for the TD than for ASD participant, while no significant differences were found for the least and most recognizable as a face images 1 and 10. Images 1 and 10 are taken from Pavlova et al.^30^, PLoS One, Creative Commons Attribution (CC-BY) license.

The stimuli consisted of ten Face-n-Food images composed of food ingredients (fruits, vegetables, sausages, etc.) that resembled faces to a different degree (Figs. 1 and 2). The Face-n-Food paradigm is described in detail elsewhere^30^. In brief, participants were presented with a set of images, one by one, in the predetermined order from the least to most resembling a face (images 1 to 10). This order was determined earlier with adult TD volunteers. This fixed order had been used, because once seen as a face, Face-n-Food images are often processed with a strong face-dominating bias. On each trial, participants had to perform a spontaneous recognition task. No immediate feedback was provided. In the present study, a computer version of the task was used. Each image was displayed on a 1024 × 768 resolution 52 × 29 cm HP V241A monitor screen. The image size subtended 11.57° × 14.43° of visual angle, and it was seen against a white background. Participants were seated at an observation distance of 60 cm from the computer screen displaying the stimuli.

#### Apparatus

 For stimuli presentation and recording of eye gaze, the EyeLink 1000 Plus eye tracker system (SR Research Ltd., Ottawa, ON, Canada) with desktop mount and SR Research Experiment Builder software (Version 2.1.140) was used. The device was in a remote mode with the head free to move with monocular tracking at temporal resolution of 500 Hz, spatial accuracy of 0.25°–0.50° visual angle, and a spatial resolution of 0.05°. Fixation data were extracted with SR Research EyeLink Data Viewer Software (Version 3.2.48) and exported to a LibreOffice spreadsheet. The regions of interest (ROIs) in the image were positioned to resemble the distributions of the features of a human face: a face was represented as an ellipse of 943,942-pixel area, containing a rectangular mouth of 12,180-pixel area, and the right and left eyes, rectangles of respectively 5548 and 5175 pixels (Fig. 1). Each image included a ROI of the same size for the left/right eyes, mouth, and face; from one image to the other, their positions varied only slightly. To ensure that a whole feature area was captured, for each face feature (e.g., eyes) the largest area across all images had been chosen.

### Procedure

Prior to examination, a proper calibration was performed by using the Eyelink 9-point grid calibration routine procedure. The calibration target was a filled black circle of 16-pixel diameter with an inner white 4-pixel diameter circle. Following calibration, participants were presented with a set of ten images, one by one, in the predetermined order from the least to most resembling a face (images 1 to 10). After each image, a blank white screen was presented, followed by a central target point for drift correction, which measured the difference between the fixation position and the target. On each trial, participants had to perform a spontaneous recognition task: they were asked to briefly describe what they saw. No immediate feedback was provided regarding performance. To avoid time pressure that can potentially cause stress and negative emotional and physiological reactions blocking cognitive processes, there was no time limit on the task. With each participant, the testing procedure lasted no longer than 20–25 min.

To examine the spatial distribution of eye gaze, the stimuli were divided into five ROIs: Face, Eyes (as a sum of the proportion of time spent on the left and right eye), Mouth, CFA (a face area beyond the eyes and a mouth), and Non-Face (an area outside a face). Considering the slight variability of the size of the face features, we defined region of interest separately for each image. For each participant (*i* = 1, …, 32) and for each image $\:{P}_{l}$ (*l* = 1, …, 10), we computed the proportion ($\:{f}_{i{j}_{{P}_{l}}}$) of the time spent looking at each area of interest *j* (*j* = 1, …, 5), which refers to the amount of time spent on a ROI divided by the total time spent on that image. In detail:

$$\:{f}_{i{Face}_{{P}_{l}}}={f}_{i{Eyes}_{{P}_{l}}}+{f}_{i{Mouth}_{{P}_{l}}}+{f}_{i{CFA}_{{P}_{l}}}$$

and

$$\:{f}_{i{Face}_{{P}_{l}}}+{f}_{i{NON-Face}_{{P}_{l}}}=1$$

In fact, $\:{f}_{i{Face}_{{P}_{l}}}$ complements $\:{f}_{i{NON-Face}_{{P}_{l}}}$ to 1. Therefore, when one evaluates Face ROI, the Non-face ROI is evaluated implicitly. That allowed to reduce the number of ROIs from five to four (Face, Eyes, Mouth, and CFA).

### Statistical analysis

For each of 32 participants the following variables were collected: age, ASD (Yes/No), and biological sex (F/M). For each image P_*l*_ (with *l* = 1, …,10), we introduced a dummy variable: 1 if a participant identified an image as a face and 0 otherwise; the number of images identified as a face ranged from 0 to 10. For each image, response time (RT, in milliseconds), in our case, the time that each participant spent to view and briefly name the image (e.g., face, food) was recorded. To detect differences in RT from image 1 to 10 in the TD control and ASD groups, we applied the Friedman rank sum test. There were no missing responses.

As quantitative variables, mean, standard deviation (SD), median, 95%, and range (min-max) were used. The Mann-Whitney *U* (Wilcoxon rank sum) test was performed to compare the distribution of the variables across the two groups. As qualitative variables, the absolute frequencies for each category (face, no face) had been compared with the Fisher’s exact test.

As for each region of interest (ROIs: *Face*,* Eyes*,* Mouth*, and *CFA*), the viewing time for single images had been measured, we needed a method to synthetize the data obtained. For this purpose, machine learning algorithms were employed. For each ROI separately, we performed a Principal Component Analysis (PCA) using as input variables the proportions of time spent looking at the respective ROI across images 1 through 10. From each of the four PCA, the first Principal Component (PC_*j*_ where *j* = 1,…,4 denotes the ROIs above) was extracted that represented a linear combination of the ten measures (with weights) in a unique synthetic indicator. The use of PCA was motivated by the intention to apply an unsupervised method (i.e., one that unlike multivariate models, does not consider an outcome) capable of producing a unique indicator (specifically, the first PC) for single ROIs. This indicator simultaneously accounts for the time spent on each of the 10 images, with each time value weighted proportionally to its relative contribution to the overall variability of the indicator. The quantitative variables (PC_*j*_ computed for each ROI: PC on face, PC on mouth, PC on eyes, and PC on CFA), stratified according to the two groups, had been visualized by means of boxplots where the title reports the *p*-value of the Wilcoxon rank sum (Mann-Whitney) tests, given that data were not normally distributed (according to the Shapiro-Wilk test).

We modelled the number of images identified as a face (*y*) by means of Generalized Linear Models (GLM) with a Poisson family distribution (well-suited in the case of count outcome), where the covariates were the diagnosis, the PC_*j*_, and the interaction term ($\:{PC}_{j}\cdot\:Diagnosis$). In detail:

$$glm\left( {y\sim Diagnosis + PC_{j} + PC_{j} \cdot Diagnosis,family = Poisson} \right)~with~j = 1, \ldots ,4$$

In this way, we generate one different model for each ROI, obtaining 4 models.

In order to obtain a ranking from the most to the least important PC_*j*_ in predicting the number of images identified as a face, we used the relative variable importance measure (relative Mean Decrease in Accuracy, rMDA) extracted from the Random Forest algorithm^38^, where we have grown 10,000 regression trees with *y* as the outcome and the PCs as covariates. In detail:

$$randomForest\left( {y\sim PC_{{Face}} + PC_{{Eyes}} + PC_{{Mouth}} + PC_{{CFA}} ,ntrees = 10,000,type = regression} \right)$$

The rMDA ranges from a minimum of 0 (when in the model, there are uninformative variables) to a maximum of 100 when the model has several predictors varying in their power.

For better understanding the relationship between *y* and the covariates, we also ran the Partial Dependence Plot (PDP) which shows the marginal effect of the covariates (one at a time) on the predicted outcome. Consequently, a PDP can show whether the relationship between *y* and the PCs is linear, monotonic or more complex.

Finally, we repeated the same procedure for understanding which PC_*j*_ impact more on the prediction of the diagnosis (TD/ASD). In this case, the rMDA measure was extracted from a Random Forest of 10,000 classification trees where the Diagnosis was the outcome and the PCs were the covariates:

$$\:Diagnosis\:randomForest\left({PC}_{Face}+{PC}_{Eyes}+{PC}_{Mouth}+{PC}_{CFA},ntrees=\text{10,000},type=classif\right)$$

Statistical data processing was performed using R software (version 4.4.2).

## Results

### Behavioral data: recognition

Behavioral data were partly reported earlier^16^. In brief, thresholds for recognition of face-pareidolia (Face-n-Food) images as a face in ASD individuals were substantially higher than in TD controls. Individuals with ASD often did not report seeing a face on the images easily recognized as a face by TD controls and gave on overall fewer face responses. Here, we re-analyzed the behavioral data by using a GLM model where the dependent variable was the number of images recognized as a face and the diagnosis served as a covariate. As a family distribution, we adopted the Poisson. For the ASD data, we expressed the coefficients by the exponential function, using the TD data as the reference and obtaining 0.706 (*p* = 0.020). ASD individuals identified as faces about 30% fewer face-pareidolia images than TD controls.

No significant differences between ASD and TD individuals were observed for the first four (N°1, 2, 3, and 4) and the last three (N° 8, 9, and 10) images. However, the intermediate images N° 5, 6, and 7 elicited substantially fewer face responses in ASD than in TD individuals (image 5: *p* = 0.003, image 6: *p* = 0.014; image 7: *p* = 0.033).

### Behavioral data: response time

For each image, we analyzed response time (RT) comparing TD against ASD individuals across all images. TD controls (light-blue boxplots, Supplementary Materials, Fig. S.1) exhibited gradually declining RT in accord with image resemblance as a face (*chi-squared χ*^2^ = 45.77, *p <* 0.001; here and throughout the work two-tailed). By contrast, in the ASD group (red boxplots, Supplementary Materials, Fig. S.1), RT did not significantly change across the images 1 through 10, and no reduction in RT occurred even for the image 10 most resembling a face (*χ*^2^ = 8.72, *p =* 0.46).

### Visual scanning strategies

The present work was focused primarily on the visual strategies applied by ASD and TD individuals for face encoding. In order to examine the spatial distribution of eye gaze, for each image we computed the summary statistics on proportion of time spent looking at the four regions of interest (ROIs: Face, Eyes, Mouth, and CFA) separately for the two groups (Table S.1). The proportion of time spent looking at a face (image 5: *p* = 0.028, and 7: *p* = 0.05) and the eyes (image 9: *p* = 0.018) are significantly greater for TD than for ASD individuals. No significant difference was found for images 6, 8, and 10. Figure 2 represents an example of visual fixation map in a participant with ASD (N6) and in a TD (N9) control.

The proportions of time spent looking at ten different images ($\:{f}_{i{j}_{{P}_{l}}}$) were used for the PCA and the PCs extracted for each ROI *j*. In Supplementary Materials, the results obtained from each PCA are given. Tables S.2 and S.3 report the proportion of variance explained and the loadings of each first PC_*j*_, respectively. As seen in Table S.2, PC_*FACE*_ attains the highest proportion of variance explained ($\:\cong\:70\%$).

**Fig. 3:**
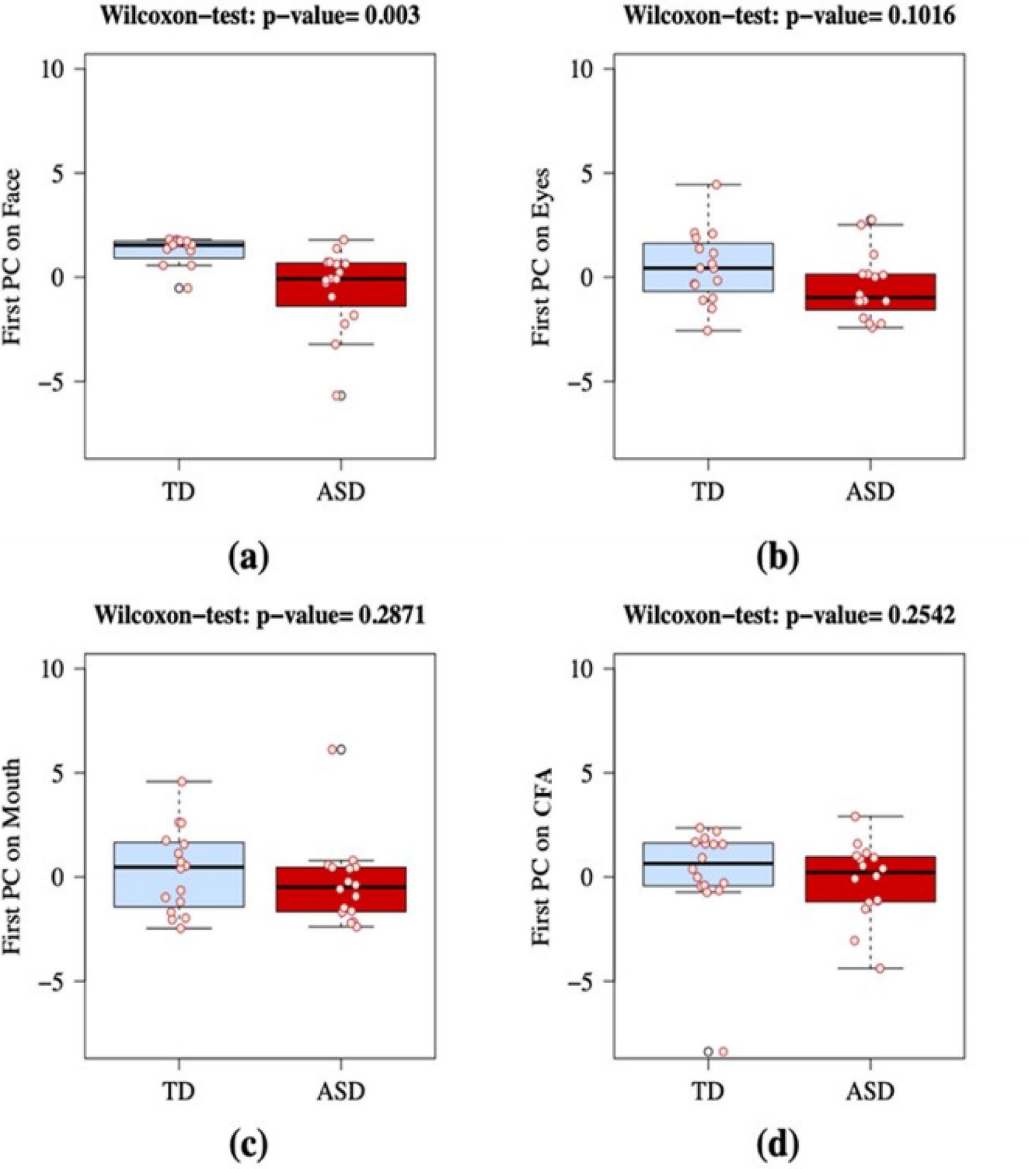
Boxplots for four PCj (ROI-specific loads) in TD (left in each plot, blue) and ASD (right in each plot, red) individuals: (**a**) Face, (**b**) Eyes, (**c**) Mouth, and (**d**) Complementary face area, CFA. For group comparisons, two-tailed Wilcoxon rank sum (Mann-Whitney) tests were used throughout.

For each ROI, we applied a GLM model, where the dependent variable *y* is the number of images identified as a face and the covariates are the diagnosis, first PC_*j*_ (ROI-specific load based on looking time), and interaction term ($\:{PC}_{j}\cdot\:Diagnosis$). We expressed the coefficients with the exponential function, and the reference category for the variable Diagnosis was TD. Table S.4 (Supplementary Materials) reports the results of the four GLMs. In all ROIs, the unique covariate that predicts the number of face responses is the diagnosis. By contrast, PCs and PC by Diagnosis interaction do not contribute to the prediction of face responses (all *p*-values are > 0.05). PCs have higher values for TD individuals (Fig. 3).

TD individuals looked longer at all four areas analyzed, but this difference was significant for the Face area only (Fig. 3a, Wilcoxon rank sum test, *W* = 205, *p* = 0.003), indicating that compared to TD, ASD individuals spent relatively less time fixating the Face area.

As the next step, we analyzed which of PCs had more pronounced impact on face identification. With this purpose in mind, we performed the Random Forest analysis, where face identification (*y*) was predicted by the four PCs, and then extracted the relative variable importance measure (rMDA). Figure S.2 (Supplementary Materials) shows a ranking from the most to the least important PC_*j*_ in predicting face identification (*y*) with a maximum of 100. The PCAs indicate that time spent looking at the mouth is the most powerful predictor of face identification. Yet this analysis does not indicate how (negatively or positively) looking time is related to face identification.

While determining overall predictor importance is a crucial task in any supervised machine learning problem, the ranking reflects only a part of the story, and often it is necessary to assess the relationship between the response *y* and the covariates. This can be done by constructing Partial Dependence Plots (PDPs). In our case, they help to visualize the relationship between *y* (the number of images identified as faces) and a PC_*j*_ (ROI-specific loads) while accounting for the average effect of the other covariates in the model.

Figure S.3 (Supplementary Materials) reports the PDPs, one for each PC_*j*_. These plots show that the longer participants look at the Mouth area and CFA, the fewer images they identify as a face (Figures S.3ab). On the contrary, the longer they look at the Face and Eyes areas, the more frequently they identify an image as a face.

**Fig. 4:**
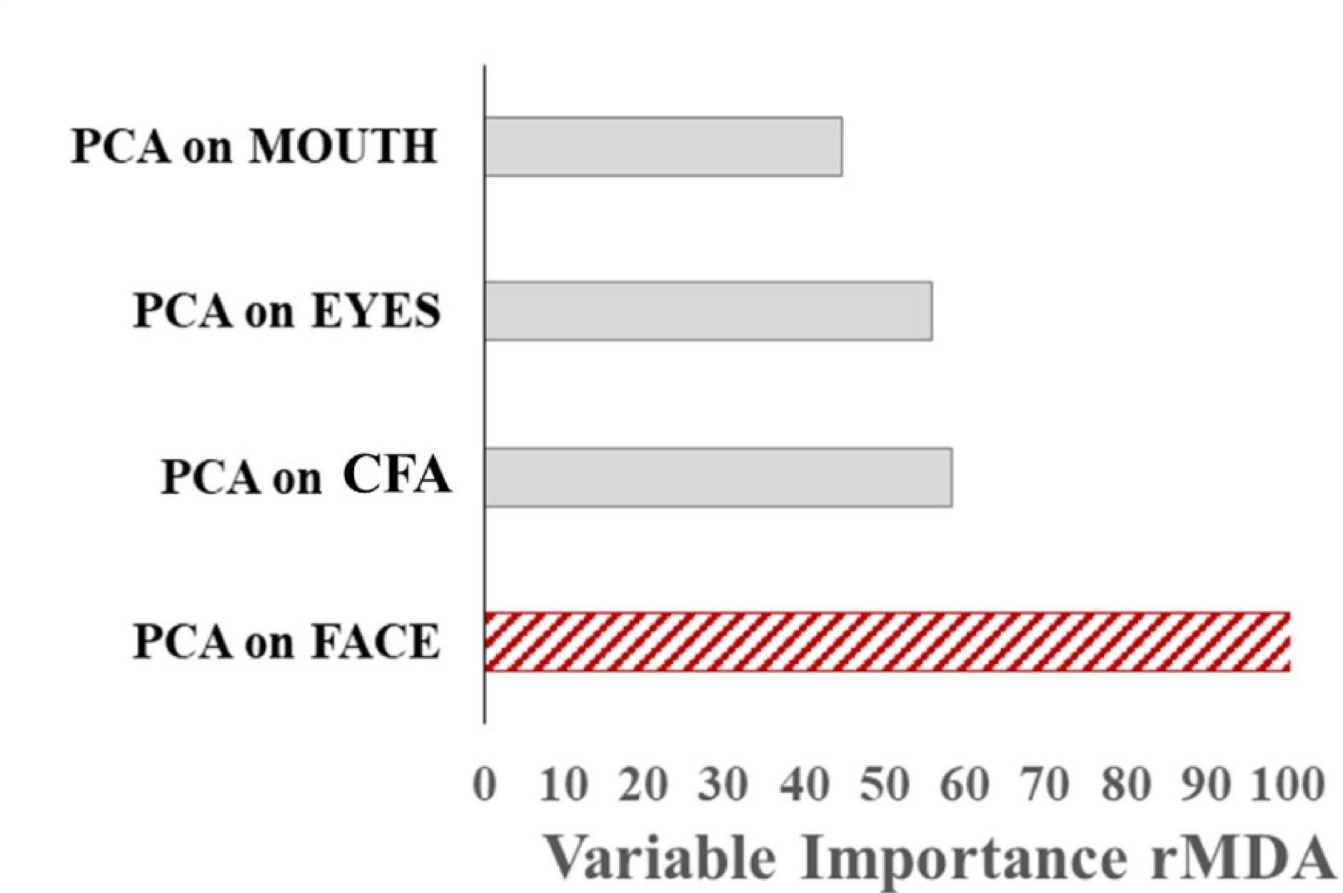
Variable importance measure rMDA extracted from a Random Forest analysis where the diagnosis is predicted by the four PC_*j*_. For the ASD and TD groups pooled together, the most powerful predictor of diagnosis is PCA on face.

In the final Random Forest analysis, we asked which of the PCs in this study could be useful in prediction of the diagnosis. To this end, we ran a model, in which the outcome was the diagnosis and the covariates were the four PCs (ROI-specific loads). In detail, we grew 10,000 classification trees (dummy variable, TD = 0 and ASD = 1), extracting the rMDA. As seen from (Fig. 4), the most powerful PC_*j*_ capable of predicting the diagnosis stems from the PCA on face.

**Fig. 5:**
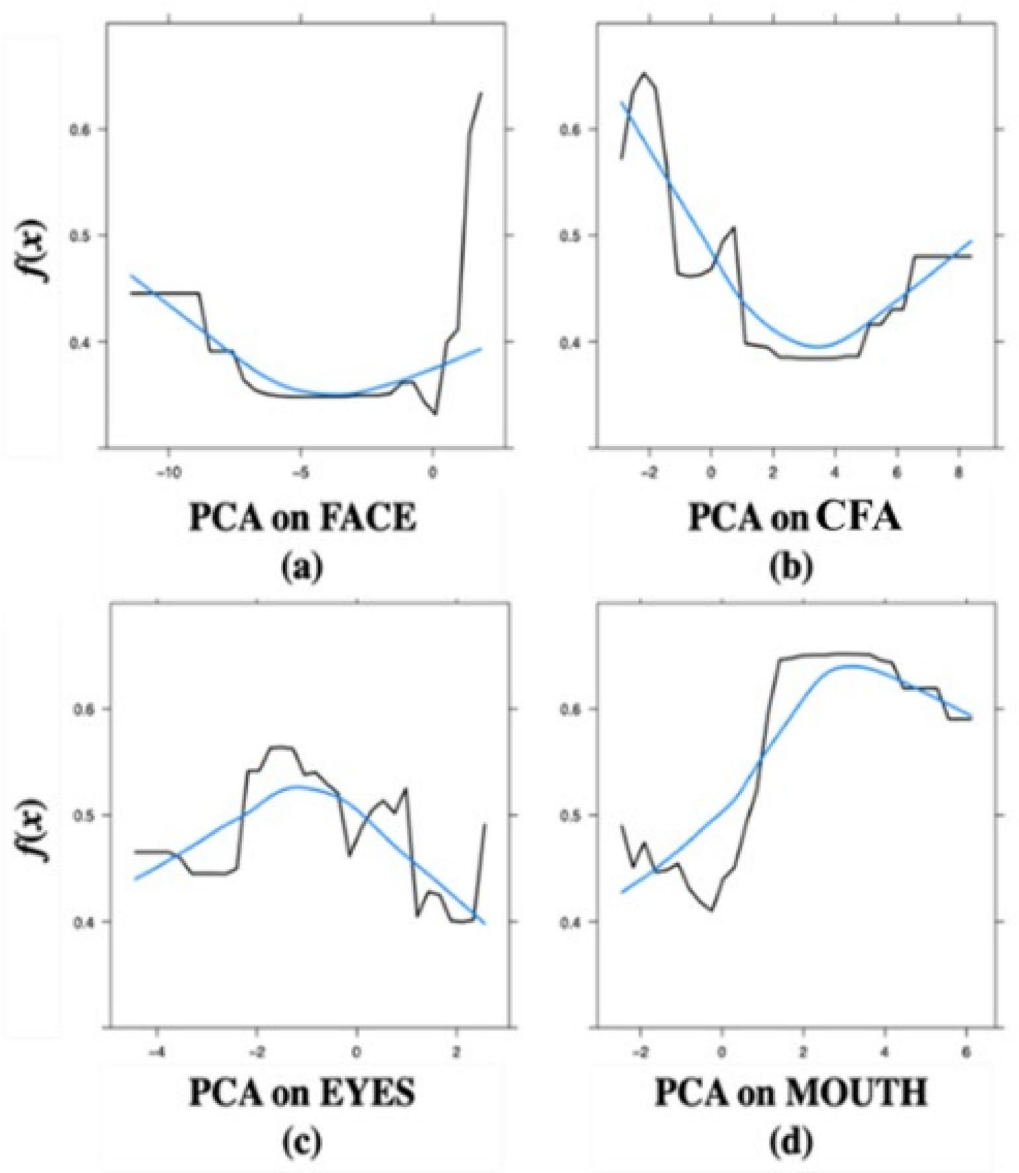
PDPs from the Random Forest analysis for the ASD and TD groups pooled together, in which the diagnosis is predicted by the PC_*j*_ (ROI-specific loads): (**a**) Face, (**b**) CFA, (**c**) Eyes, and (**d**) Mouth. PCA scales are based on time spent looking at specific ROIs. Blue curves represent the smoothing of the function.

In the PDPs shown in Fig. 5, we visualized the relationship between the Diagnosis and the PCs (once at a time). As seen in Fig. 5, as the time spent looking at the Mouth area increases, the probability of being classified as having ASD increases as well. The same holds for the CFA. By contrast, as the time spent looking at the Eyes area increases, the probability of being classified as having ASD decreases. Finally, as the time spent on the Face area increases, the probability of being classified as having ASD decreases (becoming almost constant from PCA_*FACE*_ > −3).

## Discussion

The present study aimed at exploring the issue of whether deficient face tuning in face-pareidolia images in ASD is accompanied by alterations in visual scanning strategies. The outcome indicates that higher thresholds for face tuning are associated with atypical visual scanning strategies in ASD individuals. Methodologically, the innovative aspect of this research is the use of face-pareidolia non-face images that do not contain usual face features such as a nose or a mouth.

In accord with earlier reports and analyses^16,32^, adolescents with and without ASD were found to experience substantial difficulties in spontaneous recognition of the first four images of the Face-n-Food paradigm while they experience less trouble in identifying as a face the last three ones. The Face-n-Food paradigm consists of ten images presented in a predetermined order from the least to most resembling a face in such a way that the first images are difficult to recognize as a face even for healthy adult participants^30^, while the last image elicits a face impression even in individuals with neurodevelopmental disorders such as Williams syndrome^39^ or with mental conditions such as major depressive disorder^33^ or paranoid schizophrenia^32^. A substantial difference between ASD and TD individuals occurred during viewing the intermediate images: ASD adolescents exhibited a face-tuning inferiority compared with TD controls.

In addition, TD individuals gradually reduced response time according to the increase in face resemblance, while in ASD individuals no such reduction in response time was found. Previous findings on face recognition in neurotypical adults reveal that optimal face identification is achieved with two fixations, and additional viewing time does not improve performance^40^. These findings are in agreement with our outcome: TD individuals showed short response time probably because they required only a few fixations for face recognition. Conversely, individuals with ASD seem to have difficulties in speedy face processing^41^.

For better understanding the origins of this deficit, we implemented an eye tracking methodology. The proportion of time spent looking at the Non-Face area was significantly higher for ASD adolescents as compared to TD controls, especially when viewing the intermediate images of the Face-n-Food paradigm (images 5–7). The atypical visual scanning may, at least partly, account for the difficulties in face identification experienced by ASD adolescents. Consistent with previous eye tracking studies, our work revealed that characteristics of stimuli could influence the visual scanning strategies^25,42,43^, and the capability to recognize these stimuli^24^. Specifically, the impairment of the face recognition system is more evident when the paradigms require a fine-grained perceptual discrimination (as in the case of the images less recognizable as a face) rather than a more basic perceptual skill (as in the case of the most recognizable image 10)^44^.

Most importantly, we found that the longer participants look at the Mouth and CFA regions, the lower the probability with which they report face pareidolia. Conversely, the longer they look at the Eyes region, the higher the probability that they identify an image as a face. In other words, ASD adolescents identify fewer images as a face than TD controls do and, accordingly, they spend more time fixating the Mouth and CFA regions, while TD controls look longer at the Eyes area.

Data on the distribution of visual attention during the processing of static faces in individuals with ASD are inconsistent: while several studies document decreased fixation time for the eye region and increased fixation time for the mouth area compared to TD controls^27,44–48^, there is also evidence suggesting visual scanning similarity between ASD and TD individuals^49,50^. The present findings dovetail with both the *eye avoidance*^51,52^ and the *eye indifference* hypotheses^53^. According to these assumptions, ASD individuals concentrate less on the eyes presumably either because they represent a source of emotional information that make them feel uncomfortable and socially threatened (*eye avoidance*)^51^ or because they are not sufficiently engaging or adaptively informative (*eye indifference*)^53^. Therefore, their gaze is more often directed at the mouth area and to external features as a compensatory strategy to reduce the discomfort (*eye avoidance*)^51^ or as preference for non-social stimuli (*eye indifference*)^53^.

In agreement with this, we found that the more the visual fixation focused on the mouth or areas beyond a face, the more probable is ASD occurrence. Infants with ASD show a decline in eye fixation within the first 2 to 6 months of life followed by a preferential looking toward the mouth or objects^54^. This visual scanning strategy could exert a negative impact on subsequent social development^54^. In general, altered direct gaze processing negatively impacts mutual gaze, mother-infant bonding, and shared and joint attention^55^. Moreover, the interaction between the joint effect of atypical social experience accumulated from early infancy and pathogenic factors responsible for ASD could determine potential developmental changes that shape the individual outcome^54^. Finally, another possible account for less engagement of individuals with ASD with the eyes could be the lack of the non-specific ‘top-heavy’ bias, which is defined as the tendency to focus more on elements in the upper part of a configuration or pattern^56–58^. In general, the outcome of the present study aligns with the eye-avoidance hypothesis, eye indifference hypothesis, and the lack of non-specific top-heavy bias hypothesis.

One of the limitations of the present study is the small sample size that may affect generalization of the findings. Second, as our sample consisted of ASD males (except one female), special examination is required of female individuals with ASD. ASD is known to be a gender (social construct reflecting social roles, norms and expectations)/sex (neurobiological construct)-specific neurodevelopmental disorder primarily affecting males. Our findings, therefore, may reflect the features of male ASD. Third, we examined only individuals with high-functioning ASD that demonstrated intact initial eye gaze to faces, excluding persons with more severe functional impairments that may show more pronounced alterations and difficulties in eye fixation.

In a nutshell, the present findings suggest that lower face tuning to face-pareidolia images (that do not contain habitual face cues) in adolescents with ASD is accompanied by atypical face-scanning patterns focused either on a mouth area or external areas outside of the image content. Moreover, ASD individuals less looked at the eyes area compared to their TD peers. This visual strategy, on one hand, could lead to social and emotional problems since social interaction strongly depends on interpretation of facial/gaze signals. On the other hand, the ‘eye avoiding’ strategy may serve as an adaptive response that protects individuals with ASD from the discomfort that may be generated by the eye contact. Finally, our findings suggest that visual fixation either on the atypical face-resembling elements such as the mouth or areas outside a face-like image could serve as a potential biomarker, at least one of them, for a proper ASD diagnosis.

## Electronic supplementary material

Below is the link to the electronic supplementary material.

Supplementary Material 1

## Data Availability

The data that support the findings of this study are available from the corresponding author upon reasonable request.
