# Supplementary material for: Alterations in looking at face-pareidolia images in autism
Source: Sci Rep. 2025 Apr 28;15:14915. doi: 10.1038/s41598-025-98461-7 (PMC12038013; doi:10.1038/s41598-025-98461-7)
Supplement: Supplementary file 1 — Supplementary Material 1 [file 41598_2025_98461_MOESM1_ESM.docx]

**Alterations in looking at face pareidolia images in autism**

Jessica Galli^1,2*^, Marika Vezzoli^3^, Erika Loi^1,2^, Serena Micheletti^2^, Anna Molinaro^1,2^, Lucia Tagliavento^2^, Stefano Calza^3^, Alexander N. Sokolov^4^, Marina A. Pavlova^4†^ and Elisa M. Fazzi^1,2†^

^1^ Department of Clinical and Experimental Sciences, University of Brescia, Brescia, Italy

^2^ Unit of Child Neurology and Psychiatry, ASST Spedali Civili of Brescia, Brescia, Italy

^3^ BDbiomed, BODaI Lab, University of Brescia, Brescia, Italy

^4^ Social Neuroscience Unit, Department of Psychiatry and Psychotherapy, Tübingen Center for Mental Health (TüCMH), Medical School and University Hospital, Eberhard Karls University of Tübingen, Tübingen 72076, Germany

*Correspondence

Corresponding Author: Jessica Galli, MD, PhD, Department of Clinical and Experimental Sciences, University of Brescia, Brescia 25123, Italy. E-mail: [jessica.galli@unibs.it](mailto:jessica.galli@unibs.it)

^†^These authors share senior authorship

**Supplementary Materials**


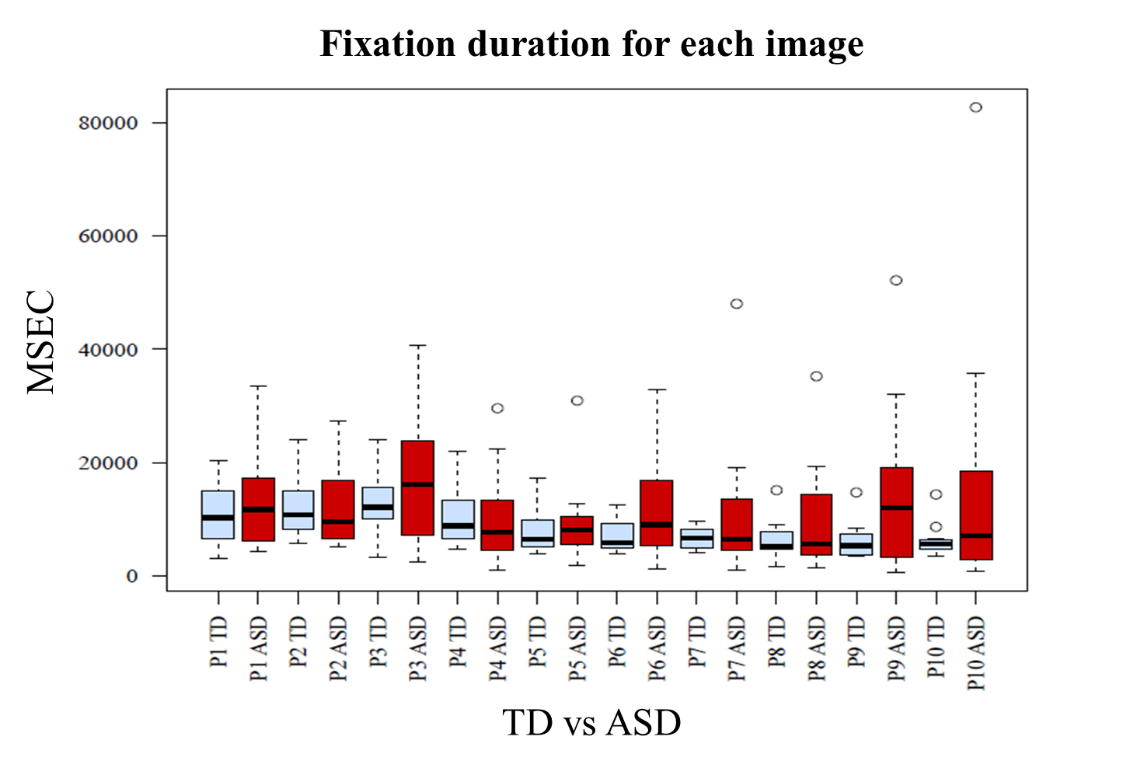


**Figure S.1.** Boxplots for the time spent on each image P_1_ – P_10_ (in msec) separately for the TD (light blue) and ASD (red) groups. The looking time significantly decreases across the ten images from the least to most resembling a face only in the TD control group (TD group, *p <* 0.001; ASD group, *p =* 0.46).


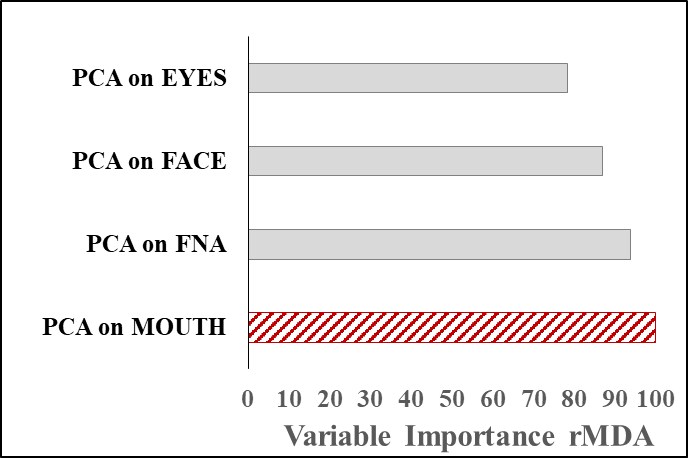

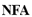

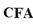


**Figure S.2.** Variable importance measure rMDA extracted from a Random Forest analysis where the number of images identified as a face is predicted by the four PC*_j_* (based on the time spent looking at each area). CFA, complementary face area. For the ASD and TD groups pooled together, the most powerful predictor of face response is provided by PCA on mouth. This does not indicate how (positively or negatively) the face response is associated with single PCAs; this is shown in **Fig. S.3**.


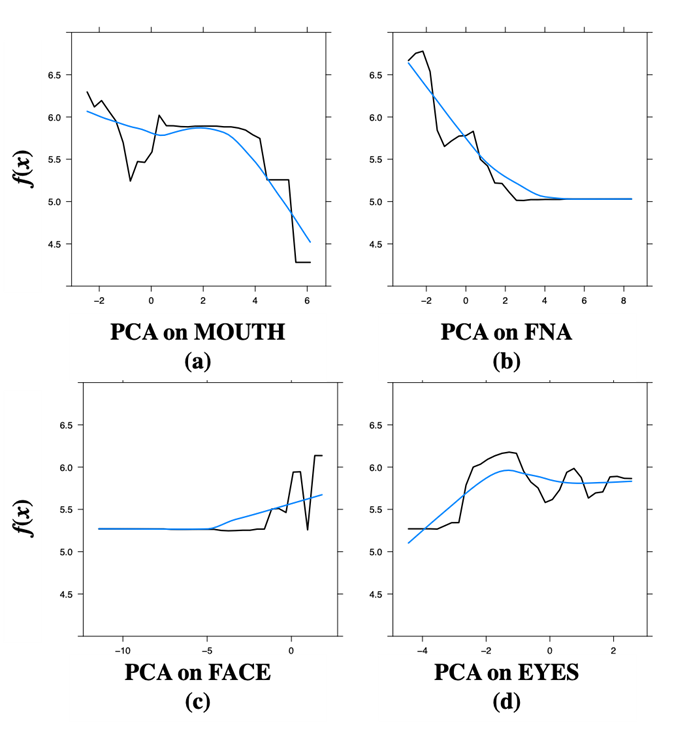

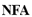

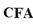


**Figure S.3.** PDPs of the Random Forest analysis for the ASD and TD groups pooled together, in which the number of images identified as faces is predicted by the PC*_j_* (ROI-specific loads): (a) Mouth, (b) CFA, (c) Face, and (d) Eyes. PCA scales are based on time spent looking at specific ROIs. Blue curves represent the smoothing of the function. The longer the participants looked either at the face or eyes areas, the more face responses they gave. By contrast, the longer the participants looked either at the mouth or CFA, the fewer face responses they provided.

**Table S.1**. Summary statistics on the proportion of time spent looking at the ROIs of each image ($f_{j_{P_{l}}}$with *j*=1,…,4 and $l$=1,…,10) both in total and stratified for the diagnosis.

| **Images** | **TD (*N*_TD_=16)** | **ASD (*N*_ASD_=16)** | **Total (*N*=32)** | ***p-value*** |
| --- | --- | --- | --- | --- |
| **Image _1_** |  |  |  |  |
| **Face** | *f_FACE_P1_* | *f_FACE_P1_* | *f_FACE_P1_* | 0.970 |
| Mean (SD) | 0.872 (0.293) | 0.869 (0.131) | 0.870 (0.223) |  |
| Median | 1.000 | 0.891 | 0.968 |  |
| Range | 0.050 - 1.000 | 0.541 - 1.000 | 0.050 - 1.000 |  |
| **Mouth** | *f_MOUTH_P1_* | *f_MOUTH_P1_* | *f_MOUTH_P1_* | 0.315 |
| Mean (SD) | 0.093 (0.102) | 0.061 (0.071) | 0.077 (0.088) |  |
| Median | 0.065 | 0.025 | 0.048 |  |
| Range | 0.000 - 0.329 | 0.000 - 0.172 | 0.000 - 0.329 |  |
| **Eyes** | *f_EYES_P1_* | *f_EYES_P1_* | *f_EYES_P1_* | 0.270 |
| Mean (SD) | 0.142 (0.093) | 0.179 (0.091) | 0.160 (0.092) |  |
| Median | 0.119 | 0.178 | 0.154 |  |
| Range | 0.000 - 0.387 | 0.043 - 0.338 | 0.000 - 0.387 |  |
| **CFA** | *f_CFA_P1_* | *f_CFA_P1_* | *f_CFA_P1_* | 0.914 |
| Mean (SD) | 0.637 (0.245) | 0.629 (0.145) | 0.633 (0.198) |  |
| Median | 0.729 | 0.659 | 0.678 |  |
| Range | 0.000 - 0.938 | 0.352 - 0.810 | 0.000 - 0.938 |  |
| **Image _2_** |  |  |  |  |
| **Face** | *f_FACE_P2_* | *f_FACE_P2_* | *f_FACE_P2_* | 0.175 |
| Mean (SD) | 0.889 (0.214) | 0.799 (0.143) | 0.844 (0.185) |  |
| Median | 0.954 | 0.843 | 0.889 |  |
| Range | 0.115 - 1.000 | 0.464 - 0.970 | 0.115 - 1.000 |  |
| **Mouth** | *f_MOUTH_P2_* | *f_MOUTH_P2_* | *f_MOUTH_P2_* | 0.495 |
| Mean (SD) | 0.050 (0.064) | 0.034 (0.066) | 0.042 (0.065) |  |
| Median | 0.019 | 0.000 | 0.010 |  |
| Range | 0.000 - 0.202 | 0.000 - 0.220 | 0.000 - 0.220 |  |
| **Eyes** | *f_EYES_P2_* | *f_EYES_P2_* | *f_EYES_P2_* | 0.829 |
| Mean (SD) | 0.127 (0.112) | 0.119 (0.099) | 0.123 (0.104) |  |
| Median | 0.118 | 0.086 | 0.105 |  |
| Range | 0.000 - 0.417 | 0.000 - 0.349 | 0.000 - 0.417 |  |
| **CFA** | *f_CFA_P2_* | *f_CFA_P2_* | *f_CFA_P2_* | 0.341 |
| Mean (SD) | 0.712 (0.199) | 0.646 (0.184) | 0.679 (0.191) |  |
| Median | 0.760 | 0.691 | 0.714 |  |
| Range | 0.060 - 0.946 | 0.197 - 0.886 | 0.060 - 0.946 |  |
| **Image 3** |  |  |  |  |
| **Face** | *f_FACE_P3_* | *f_FACE_P3_* | *f_FACE_P3_* | 0.798 |
| Mean (SD) | 0.927 (0.190) | 0.911 (0.144) | 0.919 (0.166) |  |
| Median | 1.000 | 0.983 | 0.994 |  |
| Range | 0.264 - 1.000 | 0.466 - 1.000 | 0.264 - 1.000 |  |
| **Mouth** | *f_MOUTH_P3_* | *f_MOUTH_P3_* | *f_MOUTH_P3_* | 0.159 |
| Mean (SD) | 0.104 (0.084) | 0.061 (0.083) | 0.083 (0.085) |  |
| Median | 0.115 | 0.033 | 0.053 |  |
| Range | 0.000 - 0.279 | 0.000 - 0.314 | 0.000 - 0.314 |  |
| **Eyes** | *f_EYES_P3_* | *f_EYES_P3_* | *f_EYES_P3_* | 0.689 |
| Mean (SD) | 0.205 (0.104) | 0.224 (0.154) | 0.215 (0.130) |  |
| Median | 0.196 | 0.190 | 0.192 |  |
| Range | 0.000 - 0.397 | 0.000 - 0.587 | 0.000 - 0.587 |  |
| **CFA** | *f_CFA_P3_* | *f_CFA_P3_* | *f_CFA_P3_* | 0.871 |
| Mean (SD) | 0.618 (0.159) | 0.626 (0.135) | 0.622 (0.145) |  |
| Median | 0.635 | 0.602 | 0.611 |  |
| Range | 0.244 - 0.812 | 0.413 - 0.865 | 0.244 - 0.865 |  |

*p-values <0.05 are highlighted in bold and italic*

**Table S.1 (*cont’d*).**

| **Photos** | **TD (*N*_TD_=16)** | **ASD (*N*_ASD_=16)** | **Total (*N*=32)** | ***p value*** |
| --- | --- | --- | --- | --- |
| **Image _4_** |  |  |  |  |
| **Face** | *f_FACE_P4_* | *f_FACE_P4_* | *f_FACE_P4_* | 0.508 |
| Mean (SD) | 0.938 (0.200) | 0.895 (0.161) | 0.917 (0.180) |  |
| Median | 1.000 | 0.953 | 0.983 |  |
| Range | 0.193 - 1.000 | 0.408 - 1.000 | 0.193 - 1.000 |  |
| **Mouth** | *f_MOUTH_P4_* | *f_MOUTH_P4_* | *f_MOUTH_P4_* | 0.682 |
| Mean (SD) | 0.155 (0.149) | 0.135 (0.135) | 0.145 (0.140) |  |
| Median | 0.118 | 0.124 | 0.124 |  |
| Range | 0.000 - 0.547 | 0.000 - 0.380 | 0.000 - 0.547 |  |
| **Eyes** | *f_EYES_P4_* | *f_EYES_P4_* | *f_EYES_P4_* | 0.122 |
| Mean (SD) | 0.267 (0.143) | 0.184 (0.150) | 0.226 (0.150) |  |
| Median | 0.274 | 0.176 | 0.214 |  |
| Range | 0.015 - 0.583 | 0.000 - 0.533 | 0.000 - 0.583 |  |
| **CFA** | *f_CFA_P4_* | *f_CFA_P4_* | *f_CFA_P4_* | 0.352 |
| Mean (SD) | 0.516 (0.167) | 0.576 (0.193) | 0.546 (0.180) |  |
| Median | 0.526 | 0.558 | 0.552 |  |
| Range | 0.073 - 0.824 | 0.267 - 0.994 | 0.073 - 0.994 |  |
| **Image _5_** |  |  |  |  |
| **Face** | *f_FACE_P5_* | *f_FACE_P5_* | *f_FACE_P5_* | ***0.028*** |
| Mean (SD) | 0.895 (0.150) | 0.752 (0.198) | 0.824 (0.187) |  |
| Median | 0.941 | 0.740 | 0.926 |  |
| Range | 0.435 - 1.000 | 0.378 - 1.000 | 0.378 - 1.000 |  |
| **Mouth** | *f_MOUTH_P5_* | *f_MOUTH_P5_* | *f_MOUTH_P5_* | 0.263 |
| Mean (SD) | 0.142 (0.142) | 0.095 (0.085) | 0.119 (0.117) |  |
| Median | 0.130 | 0.065 | 0.108 |  |
| Range | 0.000 - 0.550 | 0.000 - 0.294 | 0.000 - 0.550 |  |
| **Eyes** | *f_EYES_P5_* | *f_EYES_P5_* | *f_EYES_P5_* | 0.281 |
| Mean (SD) | 0.271 (0.129) | 0.216 (0.155) | 0.244 (0.143) |  |
| Median | 0.291 | 0.210 | 0.281 |  |
| Range | 0.000 - 0.470 | 0.000 - 0.460 | 0.000 - 0.470 |  |
| **CFA** | *f_CFA_P5_* | *f_CFA_P5_* | *f_CFA_P5_* | 0.469 |
| Mean (SD) | 0.481 (0.136) | 0.441 (0.173) | 0.461 (0.155) |  |
| Median | 0.474 | 0.421 | 0.469 |  |
| Range | 0.281 - 0.780 | 0.143 - 0.754 | 0.143 - 0.780 |  |
| **Image _6_** |  |  |  |  |
| **Face** | *f_FACE_P6_* | *f_FACE_P6_* | *f_FACE_P6_* | 0.311 |
| Mean (SD) | 0.950 (0.180) | 0.892 (0.135) | 0.921 (0.159) |  |
| Median | 1.000 | 0.937 | 1.000 |  |
| Range | 0.280 - 1.000 | 0.528 - 1.000 | 0.280 - 1.000 |  |
| **Mouth** | *f_MOUTH_P6_* | *f_MOUTH_P6_* | *f_MOUTH_P6_* | 0.772 |
| Mean (SD) | 0.056 (0.068) | 0.067 (0.137) | 0.061 (0.107) |  |
| Median | 0.044 | 0.017 | 0.038 |  |
| Range | 0.000 - 0.272 | 0.000 - 0.556 | 0.000 - 0.556 |  |
| **Eyes** | *f_EYES_P6_* | *f_EYES_P7_* | *f_EYES_P7_* | 0.605 |
| Mean (SD) | 0.223 (0.141) | 0.245 (0.093) | 0.234 (0.118) |  |
| Median | 0.228 | 0.254 | 0.244 |  |
| Range | 0.035 - 0.517 | 0.086 - 0.387 | 0.035 - 0.517 |  |
| **CFA** | *f_CFA_P6_* | *f_CFA_P6_* | *f_CFA_P6_* | 0.153 |
| Mean (SD) | 0.672 (0.179) | 0.581 (0.172) | 0.626 (0.179) |  |
| Median | 0.677 | 0.606 | 0.646 |  |
| Range | 0.151 - 0.885 | 0.211 - 0.847 | 0.151 - 0.885 |  |

*p-values <0.05 are highlighted in bold and italic*

**Table S.1 (*cont’d*).**

| **Photos** | **TD (*N*_TD_=16)** | **ASD (*N*_ASD_=16)** | **Total (*N*=32)** | ***p value*** |
| --- | --- | --- | --- | --- |
| **Image _7_** |  |  |  |  |
| **Face** | *f_FACE_P7_* | *f_FACE_P7_* | *f_FACE_P7_* | ***0.050*** |
| Mean (SD) | 0.940 (0.168) | 0.814 (0.183) | 0.877 (0.184) |  |
| Median | 1.000 | 0.874 | 0.972 |  |
| Range | 0.324 - 1.000 | 0.465 - 1.000 | 0.324 - 1.000 |  |
| **Mouth** | *f_MOUTH_P7_* | *f_MOUTH_P7_* | *f_MOUTH_P7_* | 0.696 |
| Mean (SD) | 0.052 (0.094) | 0.066 (0.103) | 0.059 (0.097) |  |
| Median | 0.000 | 0.012 | 0.000 |  |
| Range | 0.000 - 0.324 | 0.000 - 0.363 | 0.000 - 0.363 |  |
| **Eyes** | *f_EYES_P7_* | *f_EYES_P7_* | *f_EYES_P7_* | 0.616 |
| Mean (SD) | 0.095 (0.079) | 0.080 (0.090) | 0.087 (0.084) |  |
| Median | 0.092 | 0.062 | 0.074 |  |
| Range | 0.000 - 0.245 | 0.000 - 0.301 | 0.000 - 0.301 |  |
| **CFA** | *f_CFA_P7_* | *f_CFA_P7_* | *f_CFA_P7_* | 0.079 |
| Mean (SD) | 0.793 (0.210) | 0.668 (0.178) | 0.730 (0.202) |  |
| Median | 0.851 | 0.667 | 0.784 |  |
| Range | 0.185 - 1.000 | 0.426 - 0.913 | 0.185 - 1.000 |  |
| **Image _8_** |  |  |  |  |
| **Face** | *f_FACE_P8_* | *f_FACE_P8_* | *f_FACE_P8_* | 0.808 |
| Mean (SD) | 0.883 (0.222) | 0.868 (0.124) | 0.876 (0.177) |  |
| Median | 0.952 | 0.886 | 0.932 |  |
| Range | 0.115 - 1.000 | 0.527 - 1.000 | 0.115 - 1.000 |  |
| **Mouth** | *f_MOUTH_P8_* | *f_MOUTH_P8_* | *f_MOUTH_P8_* | 0.937 |
| Mean (SD) | 0.030 (0.038) | 0.031 (0.066) | 0.030 (0.053) |  |
| Median | 0.000 | 0.000 | 0.000 |  |
| Range | 0.000 - 0.110 | 0.000 - 0.263 | 0.000 - 0.263 |  |
| **Eyes** | *f_EYES_P8_* | *f_EYES_P8_* | *f_EYES_P8_* | 0.071 |
| Mean (SD) | 0.172 (0.089) | 0.102 (0.120) | 0.137 (0.110) |  |
| Median | 0.195 | 0.041 | 0.111 |  |
| Range | 0.004 - 0.334 | 0.000 - 0.338 | 0.000 - 0.338 |  |
| **CFA** | *f_CFA_P8_* | *f_CFA_P8_* | *f_CFA_P8_* | 0.461 |
| Mean (SD) | 0.682 (0.189) | 0.735 (0.210) | 0.709 (0.199) |  |
| Median | 0.707 | 0.783 | 0.729 |  |
| Range | 0.030 - 0.860 | 0.232 - 1.000 | 0.030 - 1.000 |  |
| **Image _9_** |  |  |  |  |
| **Face** | *f_FACE_P9_* | *f_FACE_P9_* | *f_FACE_P9_* | 0.229 |
| Mean (SD) | 0.914 (0.205) | 0.816 (0.244) | 0.865 (0.228) |  |
| Median | 0.983 | 0.914 | 0.958 |  |
| Range | 0.161 - 1.000 | 0.046 - 1.000 | 0.046 - 1.000 |  |
| **Mouth** | *f_MOUTH_P9_* | *f_MOUTH_P9_* | *f_MOUTH_P9_* | 0.613 |
| Mean (SD) | 0.058 (0.070) | 0.046 (0.067) | 0.052 (0.068) |  |
| Median | 0.023 | 0.014 | 0.014 |  |
| Range | 0.000 - 0.229 | 0.000 - 0.213 | 0.000 - 0.229 |  |
| **Eyes** | *f_EYES_P9_* | *f_EYES_P9_* | *f_EYES_P9_* | ***0.018*** |
| Mean (SD) | 0.170 (0.124) | 0.073 (0.090) | 0.122 (0.117) |  |
| Median | 0.230 | 0.034 | 0.091 |  |
| Range | 0.000 - 0.396 | 0.000 - 0.320 | 0.000 - 0.396 |  |
| **CFA** | *f_CFA_P9_* | *f_CFA_P9_* | *f_CFA_P9_* | 0.899 |
| Mean (SD) | 0.686 (0.239) | 0.697 (0.234) | 0.691 (0.233) |  |
| Median | 0.685 | 0.742 | 0.721 |  |
| Range | 0.066 - 0.973 | 0.018 - 1.000 | 0.018 - 1.000 |  |

*p-values <0.05 are highlighted in bold and italic*

**Table S.1 (*cont’d*)**.

| **Photos** | **TD (*N*_TD_=16)** | **ASD (*N*_ASD_=16)** | **Total (*N*=32)** | ***p value*** |
| --- | --- | --- | --- | --- |
| **Image _10_** |  |  |  |  |
| **Face** | *f_FACE_P10_* | *f_FACE_P10_* | *f_FACE_P10_* | 0.523 |
| Mean (SD) | 0.931 (0.211) | 0.893 (0.114) | 0.912 (0.168) |  |
| Median | 1.000 | 0.889 | 1.000 |  |
| Range | 0.153 - 1.000 | 0.629 - 1.000 | 0.153 - 1.000 |  |
| **Mouth** | *f_MOUTH_P10_* | *f_MOUTH_P10_* | *f_MOUTH_P10_* | 0.232 |
| Mean (SD) | 0.109 (0.099) | 0.073 (0.065) | 0.091 (0.085) |  |
| Median | 0.086 | 0.082 | 0.085 |  |
| Range | 0.000 - 0.292 | 0.000 - 0.164 | 0.000 - 0.292 |  |
| **Eyes** | *f_EYES_P10_* | *f_EYES_P10_* | *f_EYES_P10_* | 0.944 |
| Mean (SD) | 0.178 (0.125) | 0.181 (0.100) | 0.179 (0.111) |  |
| Median | 0.163 | 0.209 | 0.177 |  |
| Range | 0.000 - 0.460 | 0.000 - 0.293 | 0.000 - 0.460 |  |
| **CFA** | *f_CFA_P10_* | *f_CFA_P10_* | *f_CFA_P10_* | 0.935 |
| Mean (SD) | 0.645 (0.205) | 0.639 (0.154) | 0.642 (0.178) |  |
| Median | 0.647 | 0.607 | 0.625 |  |
| Range | 0.143 - 1.000 | 0.418 - 0.877 | 0.143 - 1.000 |  |

*p-values <0.05 are highlighted in bold and italic*

| **PC*_j_*** | **Proportion of Variance** |
| --- | --- |
| **PC*_FACE_*** | 0.715 |
| **PC*_EYES_*** | 0.285 |
| **PC*_MOUTH_*** | 0.405 |
| **PC*_CFA_*** | 0.475 |

**Table S.2.** Proportion of variance explained by each first PC*_j_* (where *j*=1,…,4 are the regions of interest) for the ASD and TD groups pooled.

**Table S.3.** Loadings of each first PC*_j_* (where *j*=1,…,4 are the regions of interest) across images P_1_ through P_10_ for the ASD and TD groups pooled.

| **Image** | **PC*_FACE_*** | **PC*_EYES_*** | **PC*_MOUTH_*** | **PC*_CFA_*** |
| --- | --- | --- | --- | --- |
| **P_1_** | 0.267 | -0.100 | 0.357 | 0.289 |
| **P_2_** | 0.348 | 0.470 | 0.383 | 0.412 |
| **P_3_** | 0.336 | 0.121 | 0.346 | 0.295 |
| **P_4_** | 0.348 | 0.215 | 0.246 | 0.258 |
| **P_5_** | 0.288 | 0.463 | 0.213 | 0.269 |
| **P_6_** | 0.358 | -0.025 | 0.359 | 0.338 |
| **P_7_** | 0.310 | 0.283 | 0.348 | 0.343 |
| **P_8_** | 0.342 | 0.246 | 0.161 | 0.380 |
| **P_9_** | 0.235 | 0.466 | 0.390 | 0.310 |
| **P_10_** | 0.307 | 0.368 | 0.268 | 0.221 |

**Table S.4.** Outcome of the four GLMs built for the number of images identified as a face. Covariates are the PC*_j_* (ROI-based loads related to time spent looking at respective areas), diagnosis, and interaction term. Coefficients are expressed with an exponential function; LCL/UCL, lower/upper 95% confidence-interval limits for respective odds ratios. The only model covariate predicting the number of face responses is the diagnosis.

| **GLM model, family=Poisson** | **Odds Ratio** | ***p*-value** |
| --- | --- | --- |
| **MODEL 1: FACE** |  |  |
| **PC*_FACE_***  **(LCL; UCL)** | 1.026  (0.965; 1.109) | 0.469 |
| **ASD vs TD (Diagnosis)**  **(LCL; UCL)** | 0.719  (0.530; 0.972) | ***0.033*** |
| **PC*_FACE_*** **\|ASD**  **(LCL; UCL)** | 0.978  (0.852; 1.128) | 0.756 |
| **MODEL 2: EYES** |  |  |
| **PC*_EYES_***  **(LCL; UCL)** | 0.986  (0.878; 1.103) | 0.808 |
| **ASD vs TD (Diagnosis)**  **(LCL; UCL)** | 0.650  (0.470; 0.890) | ***0.008*** |
| **PC*_EYES_*** \|**ASD**  **(LCL; UCL)** | 0.895  (0.734; 1.084) | 0.262 |
| **MODEL 3: MOUTH** |  |  |
| **PC*_MOUTH_***  **(LCL; UCL)** | 0.945  (0.855; 1.041) | 0.260 |
| **ASD vs TD (Diagnosis)**  **( LCL; UCL)** | 0.665  (0.488; 0.899) | ***0.009*** |
| **PC*_MOUTH_*** \|**ASD**  **(LCL; UCL)** | 0.957  (0.803; 1.126) | 0.608 |
| **MODEL 4:CFA** |  |  |
| **PC*_CFA_***  **(LCL; UCL)** | 1.053  (0.970; 1.162) | 0.261 |
| **ASD vs TD (Diagnosis)**  **(LCL; UCL)** | 0.723  (0.537; 0.970) | ***0.031*** |
| **PC*_CFA_*** \|**ASD**  **(LCL; UCL)** | 1.027  (0.874; 1.206) | 0.745 |

*p-values <0.05 are highlighted in bold and italics*
